# A Robot Mimicking Heart Motions: An *Ex-Vivo* Test Approach for Cardiac Devices

**DOI:** 10.1007/s13239-021-00566-3

**Published:** 2021-08-18

**Authors:** Adrian Zurbuchen, Aloïs Pfenniger, Sammy Omari, Tobias Reichlin, Rolf Vogel, Andreas Haeberlin

**Affiliations:** 1 Department of Cardiology, Bern University Hospital, University of Bern, Freiburgstrassse 3, 3010 Bern, Switzerland; 2 sitem Center for Translational Medicine and Biomedical Entrepreneurship, University of Bern, Bern, Switzerland; 3 ARTORG Center for Biomedical Engineering, University of Bern, Bern, Switzerland; 4 Sonceboz SA, Sonceboz, Switzerland; 5 Lyft Inc., San Francisco, CA USA; 6 Department of Cardiology, Bürgerspital Solothurn, Solothurn, Switzerland

**Keywords:** Hexapod, *Ex-vivo*, Simulator, Stewart platform, Inverse kinematic

## Abstract

**Purpose:**

The pre-clinical testing of cardiovascular implants gains increasing attention due to the complexity of novel implants and new medical device regulations. It often relies on large animal experiments that are afflicted with ethical and methodical challenges. Thus, a method for simulating physiological heart motions is desired but lacking so far.

**Methods:**

We developed a robotic platform that allows simulating the trajectory of any point of the heart (one at a time) in six degrees of freedom. It uses heart motion trajectories acquired from cardiac magnetic resonance imaging or accelero-meter data. The rotations of the six motors are calculated based on the input trajectory. A closed-loop controller drives the platform and a graphical user interface monitors the functioning and accuracy of the robot using encoder data.

**Results:**

The robotic platform can mimic physiological heart motions from large animals and humans. It offers a spherical work envelope with a radius of 29 mm, maximum acceleration of 20 m/s^2^ and maximum deflection of ±19° along all axes. The absolute mean positioning error in x-, y- and z-direction is 0.21 ±0.06, 0.31 ±0.11 and 0.17 ±0.12 mm, respectively. The absolute mean orientation error around x-, y- and z-axis (roll, pitch and yaw) is 0.24 ±0.18°, 0.23 ±0.13° and 0.18 ±0.18°, respectively.

**Conclusion:**

The novel robotic approach allows reproducing heart motions with high accuracy and repeatability. This may benefit the device development process and allows re-using previously acquired heart motion data repeatedly, thus avoiding animal trials.

**Supplementary Information:**

The online version contains supplementary material available at 10.1007/s13239-021-00566-3.

## Introduction

The global increase of cardiovascular diseases calls for more and better therapies in the future. However, novel therapeutic methods that comply with medical device regulations and provide advanced functionalities typically come at the cost of increased development complexity. Hence, accurate and realistic test environments become increasingly important.

For instance, there exist numerous cardiac devices that require an anchoring system to be secured to the heart (e.g. leadless cardiac pacemakers,^4^,^11^ pacemaker leads^17^ or prosthetic heart valves^10^). The complexity of mechanical anchoring systems increases further for valve repair procedures,^8^,^3^ where the outcome also heavily depends on the operator’s experience.^6^ Another well-known field of research focuses on developing an efficient technology to harvest energy from the beating heart via a mechano-electrical transduction mechanism to power cardiac pacemakers or other implantable devices.^19^,^13^, ^1^

Implantable devices have a broad range of applications and, thus, require various testing and training platforms.^14^ An example of the latter is a simulator for mimicking organ movements, which is used to train surgeons on surgical robots (e.g. Da Vinci™, Intuitive Surgical Inc., USA^14^). Their approach focused on a cost-effective and compact solution but suffers from limited workspace and accuracy. The anchoring of devices to myocardial tissue was analyzed by tensile testing machines that measure the maximum fixation force of a device.^10^ However, this method neglects the dynamic and cyclic nature of the heart’s motion.

Energy harvesting devices are typically tested on mechanical shaker platforms, which expose the device to unidirectional sinusoidal excitations at high frequencies (> 1000 Hz).^18^ However, a method for accurate simulation of natural heart motions *in vitro* is lacking. Thus, cardiovascular device testing and training is often performed by *in-vivo* experiments on large animal models such as pigs and sheep. Although it has been recognized that technical approaches offer desirable benefits (e.g. reproducibility, well-defined testing conditions)^2^ and that reducing the number of animal trials is desirable from an ethical viewpoint,^16^*ex-vivo* setups for mechanical testing are not yet widely used.

In this study, we introduce a novel robot, which can reproduce human or large animal heart motion *ex-vivo*. This work exemplifies what contemporary technology offers with respect to device development as it allows investigating the effect of heart motions on various types of cardiac devices.

## Methods

**Figure 1:**
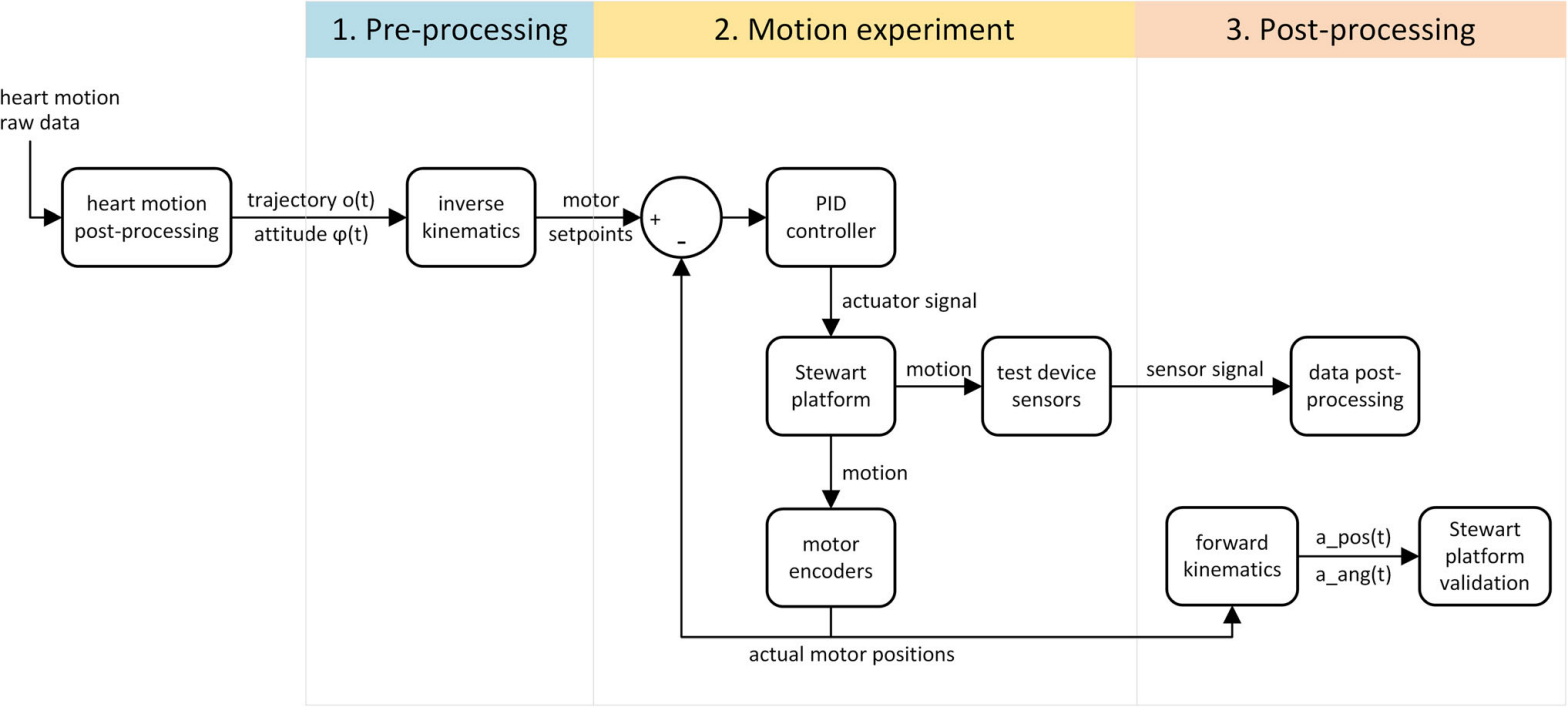
In the planning phase (blue section), the raw data of previously recorded heart motions will be translated to motor setpoints in an inverse kinematic process. These serve as input of the Stewart platform’s PID control loop during motion experiments (green section). The test subjects mounted on the Stewart platform will be exposed to repetitive heart motions. The data generated will be processed in a post-processing phase (red section). Furthermore, the Stewart platform’s motor encoder data will be used for validation.

The goal of this study was to develop a robot (Stewart platform) that mimics the motion of any point in or on the heart. This allows studying the effect of the heart’s myocardial contractions on cardiovascular devices in a repeatable manner under laboratory conditions. The robot features an actuated platform, where the devices to be analyzed can be mounted. As input, it requires three-dimensional motion trajectories and/or attitudes. Such datasets can originate from different modalities (e.g. magnetic resonance imaging, computer tomography, echocardiography or acceleration sensors). As illustrated in Fig. 1, each experiment will run through three distinctive steps: pre-processing, motion experiment and post-processing.

### Pre-Processing

**Figure 2:**
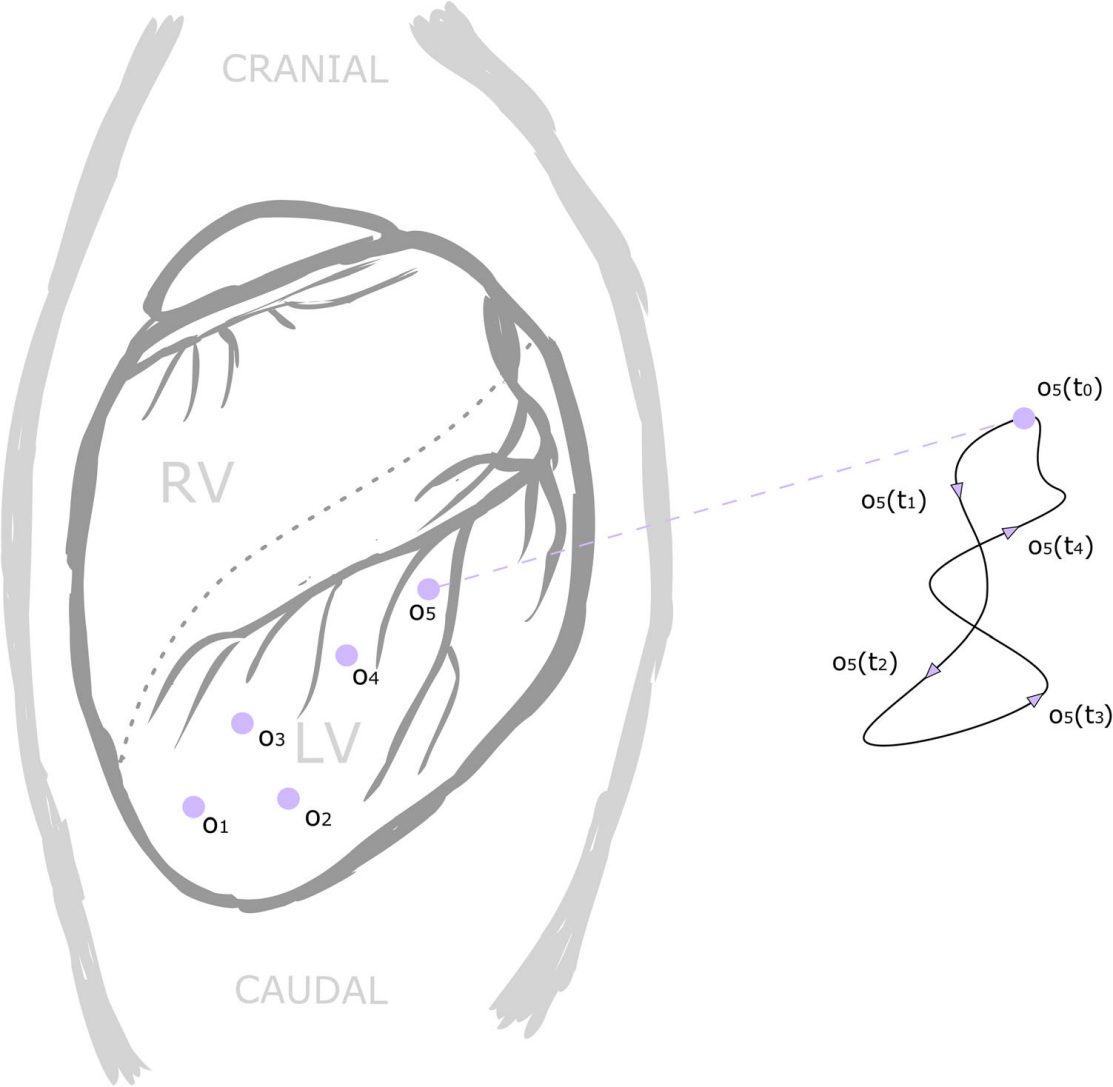
The anterior view onto the heart during an open-chest surgery. The purple points indicate the location on the heart, where time-dependent heart motion trajectories ($o_{5} \left( {t_{0} } \right)$ to $o_{5} \left( {t_{4} } \right)$) and attitude ($\phi \left( t \right)$, not shown in the figure) were acquired by means of acceleration sensors. Each pair of trajectory/attitude datasets can be used as individual input for a motion experiment with the robot.

**Figure 3:**
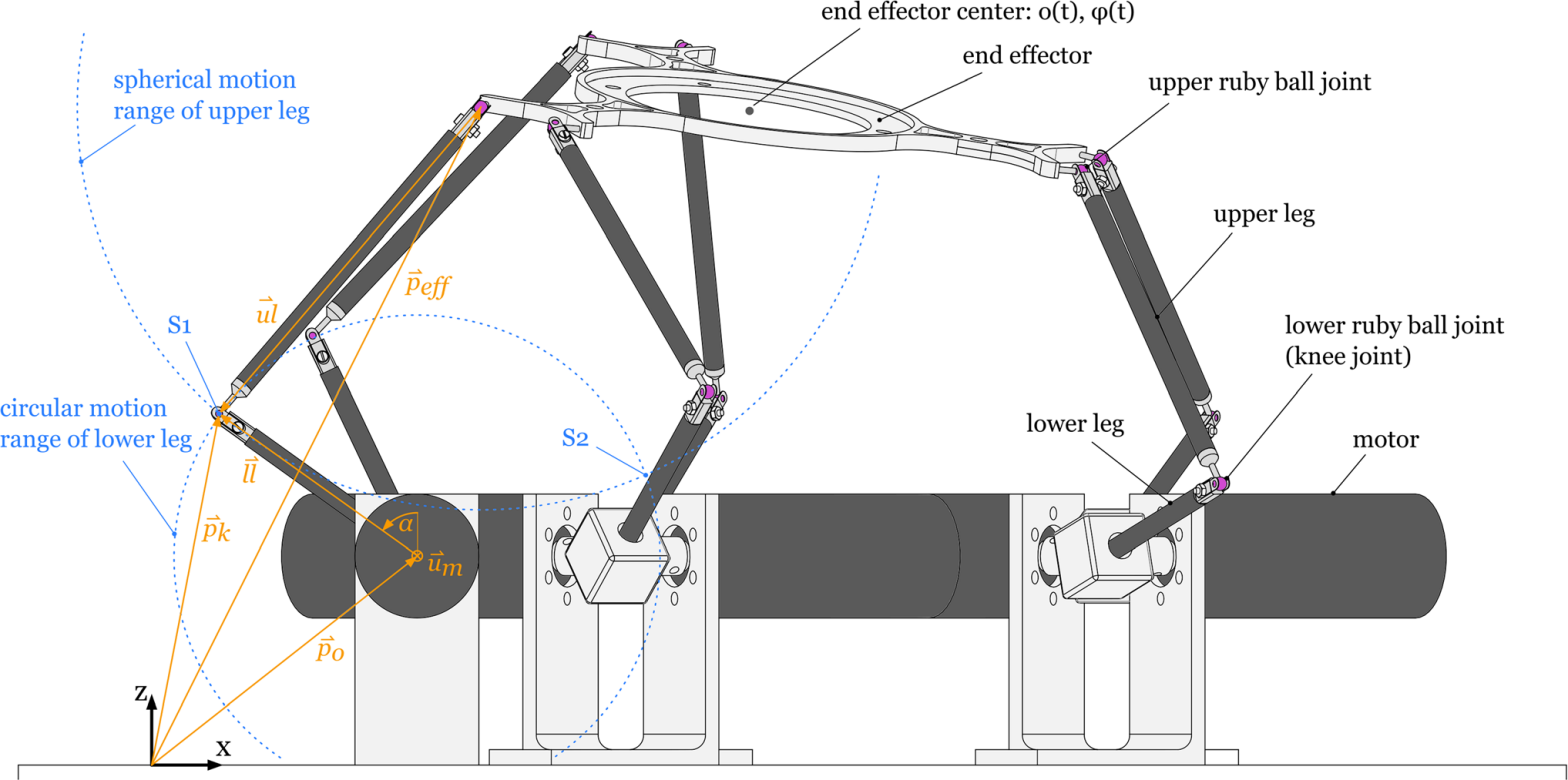
The Stewart platform showing the end effector linked to the six motors by upper and lower legs. Furthermore, the free-body diagram on the left shows position vectors to the motor origin ${\mathop{p}\limits^{\rightharpoonup}}_{o}$, knee joint ${\mathop{p}\limits^{\rightharpoonup}}_{k}$ and end-effector joint ${\mathop{p}\limits^{\rightharpoonup}}_{eff}$ and vectors for the lower and upper leg, $\mathop{ll}\limits^{\rightharpoonup}$ and $\mathop{ul}\limits^{\rightharpoonup}$, respectively. The blue circles indicate the spherical and circular motion range of the upper and lower leg, respectively. They coincide at two points, S1 and S2, which illustrates that there is no unique solution for this problem.

Heart contractions are complex 3-dimensional motion patterns that can be acquired with different methods such as acceleration sensors or magnetic resonance imaging (MRI) tagging. Typically, modality-dependent post-processing will be required, resulting in heart motion trajectories and attitudes of single points intramurally or on the endo- or epicardium (cf. Fig. 2). Based on heart motion data from previous MRI studies^20^ and inertial measurement devices,^21^ it was concluded that a robot should provide a workspace radius of 29 mm with maximal angular deflection of ± 19° to be able to mimic even extreme heart motions most realistically. In order to meet the requirements in terms of working space, degrees of freedom and dynamics, a parallel robotic structure was chosen, commonly referred to as Stewart platform. Its end effector platform is driven by six motors (cf. Fig. 3), each being linked to the platform by articulated legs. In order to move the platform along a predefined heart motion trajectory $o\left( t \right)$ and attitude $\phi \left( t \right)$, the motor angles use the following inverse kinematic process:

1. Any motion trajectory o(t) and attitude ϕ(t) of a point in/on the heart can be used to define the target position and orientation of the end effector platform over time. Thus, the position of all six joints where the legs connect to the end effector can be computed.
2. The joints at the end effector are ball joints, which allow the upper legs to rotate freely around this spherical joint. Therefore, the loose end of the upper leg can be found somewhere on a sphere with the radius of the upper leg length and its center at the spherical joint.
3. On the other hand, the lower leg is rigidly fixed perpendicular to the motor axis and can rotate only around the axis of the motor. Consequently, the other end of the lower leg can be found on a circle with center on the motor axis.
4. Since the ends of the upper and lower leg must coincide (position of the knee), the rotation angle around the motor axis can be determined by finding the intersection of the sphere with the circle (Fig. 3, S1 and S2), prescribed by the upper and lower leg, respectively.

Since this inverse kinematic process is a highly non-linear problem, it was addressed numerically. First, the position of the knee $\mathop{p}\nolimits^{\rightharpoonup} _{k}$ is calculated:

$${\mathop{p}\limits^{\rightharpoonup}}_{k} = {\mathop{p}\limits^{\rightharpoonup}}_{o} + {\mathop{ll}\limits^{\rightharpoonup}} = p_{o} + \left( {R \cdot L_{ll} \cdot {\mathop{u}\limits^{\rightharpoonup}}_{ll} } \right), = p_{o} + \left( {axisangle2rotmat\left( {\mathop{u}\nolimits^{\rightharpoonup}_{m} ,\alpha } \right) \cdot L_{ll} \cdot {\mathop{u}\limits^{\rightharpoonup}}_{ll} } \right)$$

${\mathop{p}\limits^{\rightharpoonup}}_{o}$ stands for the position of the motor axis origin and $\mathop{ll}\limits^{\rightharpoonup}$ for the lower leg vector. The latter is defined by the lower leg’s length $L_{ll}$ and unit vector ${\mathop{u}\limits^{\rightharpoonup}}_{ll}$. Furthermore, the motor axis rotation angle $\alpha$ is included either as rotation matrix $R$ or in the form of an axis-angle representation with ${\mathop{u}\limits^{\rightharpoonup}}_{m}$ and $\alpha$ as axis and angle, respectively. In a second step, we define the cost function for the numerical solver to minimize:

$$dist\left( \alpha \right) = \Vert{\mathop{p}\limits^{\rightharpoonup}}_{eff} - {\mathop{p}\limits^{\rightharpoonup}}_{k}\Vert - \Vert{\mathop{ul}\limits^{\rightharpoonup}}\Vert = \Vert{\mathop{p}\limits^{\rightharpoonup}}_{eff} - p_{o} + ( {axisangle2rotmat( {{\mathop{u}\limits^{\rightharpoonup}}_{m} ,\alpha } ) \cdot L_{ll} \cdot {\mathop{u}\limits^{\rightharpoonup}}_{ll} } )\Vert - \Vert{\mathop{ul}\limits^{\rightharpoonup}}\Vert$$

Subtracting the position vector of the knee ${\mathop{p}\limits^{\rightharpoonup}}_{k}$ from the position of the end effector ${\mathop{p}\limits^{\rightharpoonup}}_{eff}$ will result in an approximation of the upper leg vector $\mathop{ul}\limits^{\rightharpoonup}$ for an initial guess of the motor axis rotation angle $\alpha$. In addition, the function subtracts the length of the upper leg such that the numerical solver can solve for $\alpha$ that minimizes the function output ($dist\left( \alpha \right) \to 0$). Since the function has two solutions (S1 and S2 as indicated in Fig. 3) and only solution S1 is a valid option, it is crucial to approximate the initial value of $\alpha$ for the minimizing function as close as possible.

The process is repeated for all six motor-leg assemblies and for all time points of a given heart motion trajectory o(t) and attitude ϕ(t). This will result in time-dependent rotation angles for each motor. These motor setpoints serve as input for the Stewart platform’s control loop.

The angular displacements of the motors were calculated based on the input trajectory by the inverse kinematic algorithm that was implemented in Matlab (Mathworks Inc., USA) (cf. Fig. 1). The kinematics of the Stewart platform was programmed in Simulink (Mathworks Inc., USA) to simulate the motion of the platform when actuated by the motors (cf. online resource 1, video of the simulated robot). This approach allows estimating whether the physical Stewart platform can reproduce the prescribed heart motion under the given practical restrictions on e.g. workspace or joint motion range.

### Motion Experiment

**Figure 4:**
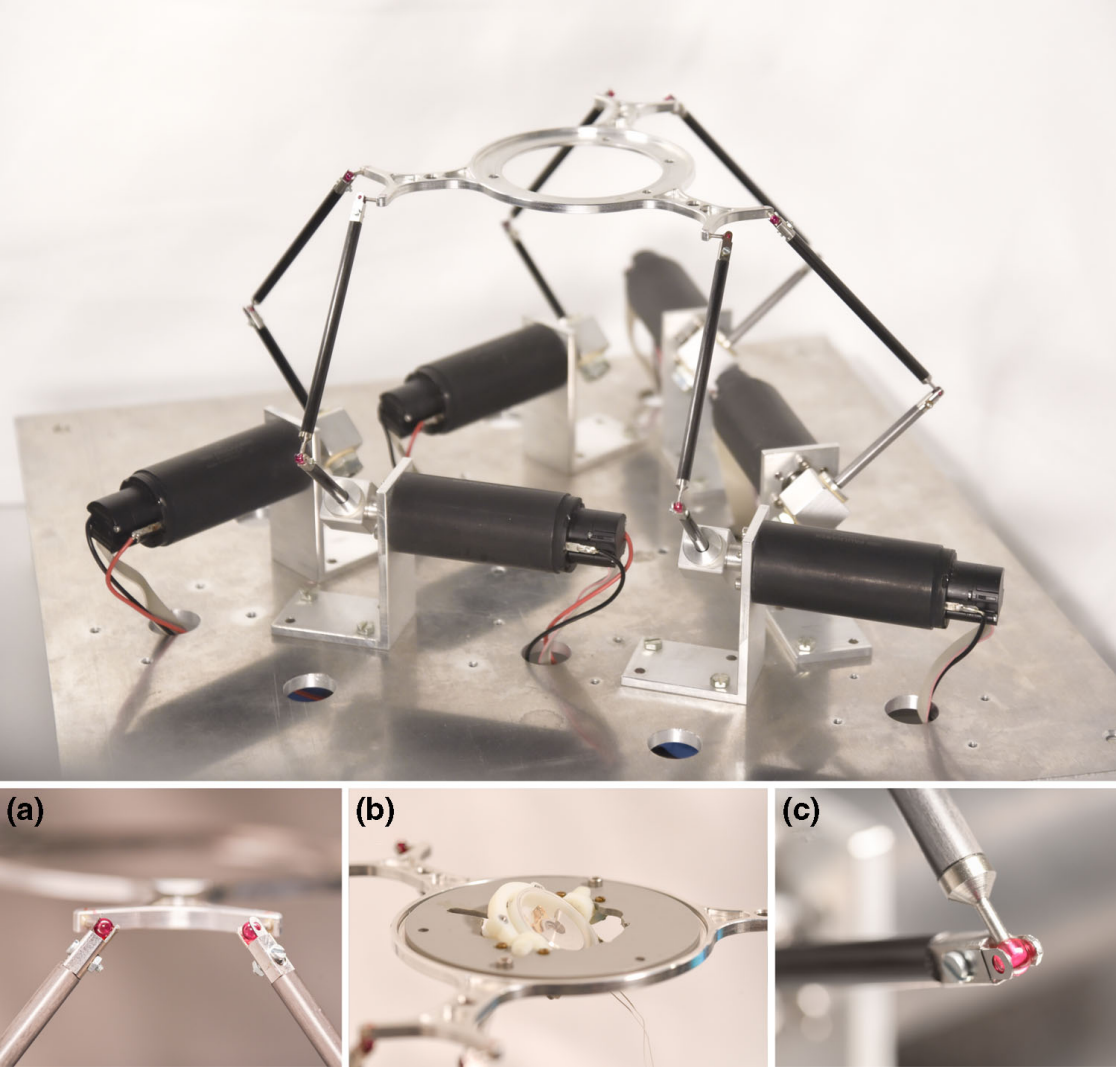
Shows the robot with the end effector platform that is connected to six motors via legs. Subpanel (a) illustrates how the ruby bearings connect two upper legs to the end effector platform. The latter features a positioning frame for the testing device and is shown in subpanel (b). The upper and lower carbon leg are connected to each other via joints that feature a fork construction to hold a ruby ball (c).

For practical illustration, three different cardiovascular energy harvesting devices were tested with the Stewart platform using a specific heart motion (AT 1c from 21). The first two candidates were epicardial energy harvesting devices, which are optimized and non-optimized prototypes for extracting energy from heart motions 21, respectively. The third device is intended for endocardial implantation.^5^ Their respective voltage outputs were measured across a 1 kΩ load resistor by a NI USB-6002 data acquisition device (National Instruments Corporation, USA). The Stewart platform performed the identical heart motion trajectory on all energy harvesting devices for a duration of 60 s. In order to measure the three different energy harvesting devices, each of them was attached to the end effector platform by a positioning frame to adjust the correct initial orientation towards gravity (Fig. 4b).

### Hardware

The Stewart platform is actuated by six motors with 10-bit encoders (3272G024CR and IE3-1024 L, Faulhaber GmbH, Germany). They are connected to Universal Motion Interfaces and a PXI controller (NI UMI-7774 and NI PXI-1031, National Instruments Corporation, USA). The motor encoders are sampled at a rate of 100 Hz. The Stewart platform was controlled by a classical closed-loop PID controller, which is a native functionality of the PXI system. A LabVIEW program serves as user interface and sends the motor setpoints ($\alpha$, derived during the inverse kinematic process), starts and stops the Stewart platform and acquires real-time values from the motors’ encoders. Furthermore, it allows recording any other voltage signal that might be generated during testing.

The Stewart platform’s aluminum end effector is 86 mm in diameter and features 6 ruby ball joints (ø 3.5 mm) to connect to the upper legs (cf. Fig. 4). The joints’ ruby balls are clamped by a fork construction, which consists of a rigid side and a flexible spring leaf (cf. Figs. 4a and 4c). The fork is designed to hold up for end effector platform accelerations of up to 20 m/s^2^ and to safely dislocate in case of collision. The same joint principle was used to build the knee joints. The joints are connected by carbon fiber tubes that form the 105 and 66 mm long upper and lower legs, respectively. The lower legs are supported by T-shaped adapters that connect the legs to the six motor axes.

In its neutral position, the Stewart platform’s end effector is at center position ($o_{x} = o_{y} = o_{z} = 0 \;{\text{mm}}$ and $\phi_{roll} = \phi_{pitch} = \phi_{yaw} = 0^\circ$) and the knee angles take a 90 degrees position. The robot reaches its lowest position when all knees are bent to 63 degrees and the knee joints are physically restricted to move further. Its highest position can be reached by stretching the legs to the upper limit of 140 degrees knee angle. The physical bending restriction of the knee joints was assessed using a 3D CAD assembly (Solidworks, USA). The stretching limit of the knee joints is not a physical restriction but rather a safety precaution to avoid positions of the end effector that would result in a non-valid kinematic solution (cf. section 0).

The Stewart platform was tested for the maximum vertical load capacity it can carry in static conditions. At the platform’s neutral position, the load was gradually increased until the motors’ overload protection would be triggered and the platform could no longer support the additional weight. This test was repeated 20 times. The Stewart platform was able to carry a mean maximum load of 442.0 g ± 16.1 g.

The hardware’s key dimensions are provided hereafter:

- Size base plate: 400 by 400 mm
- End effector outer diameter: 90 mm
- End effector weight: 33.2 g
- Lower leg length: 65 mm
- Lower leg weight including T-adapter to motor axis: 25.0 g
- Upper leg length: 104 mm
- Upper leg weight: 4.2 g

### Post-Processing

During a motion experiment, the Stewart platform encoders continuously record the motor axis position for controlling the platform in a closed-loop system in real time. After an experiment, the data can be used to verify how accurately the Stewart platform was able to reproduce a specific heart motion. In order to do that, encoder values are translated into an end-effector position in a forward kinematic process. First, the encoder values $enc\left( t \right)$ are converted into a time-dependent motor axis rotation angle $\alpha$ for each motor by

$$\alpha \left( t \right) = \frac{enc\left( t \right) \cdot 360^\circ }{{4096}},$$

where 4096 represents the encoder resolution per full revolution. Second, the motor axis rotation angles are used as input of the Simulink model that describes the kinematics of the Stewart platform. Since the end-effector is connected to the motors with a well-defined leg structure, the model calculates a unique end-effector position for any given set of motor axis rotation angles.

The actual end-effector trajectory $o_{actual} \left( t \right)$ and attitude $\phi_{actual} \left( t \right)$ acquired by forward kinematics can now be used to validate the Stewart platform with the set-point heart motion trajectory $o\left( t \right)$ and attitude $\phi \left( t \right)$. Therefore, the actual and set-point values are compared at each time step by calculating the positioning and attitude error, $err_{o} \left( t \right)$ and $err_{\phi } \left( t \right)$, respectively:

$$err_{o} \left( t \right) = \left\| {o_{{actual}} \left( t \right) - o\left( t \right)} \right\| = \left\| {\left[ {\begin{array}{*{20}c} {o_{{actual_{x} }} \left( t \right)} \\ {o_{{actual_{y} }} \left( t \right)} \\ {o_{{actual_{z} }} \left( t \right)} \\ \end{array} } \right] - \left[ {\begin{array}{*{20}c} {o_{x} \left( t \right)} \\ {o_{y} \left( t \right)} \\ {o_{z} \left( t \right)} \\ \end{array} } \right]} \right\|$$

$$err_{\phi } \left( t \right) = \left\| {\phi _{{actual}} \left( t \right) - o\left( t \right)} \right\| = \left\| {\left[ {\begin{array}{*{20}c} {\phi _{{actual_{{roll}} }} \left( t \right)} \\ {\phi _{{actual_{{pitch}} }} \left( t \right)} \\ {\phi _{{actual_{{yaw}} }} \left( t \right)} \\ \end{array} } \right] - \left[ {\begin{array}{*{20}c} {\phi _{{roll}} \left( t \right)} \\ {\phi _{{pitch}} \left( t \right)} \\ {\phi _{{yaw}} \left( t \right)} \\ \end{array} } \right]} \right\|$$

For the validation, 13 different heart motion datasets were considered, which were previously acquired^21^ by a custom-made 9-axis inertial measurement unit.

## Results

In section 0, we show that our robotic platform and associated algorithm can effectively handle the 13 available heart motion trajectories. The complete working Stewart platform can be seen in a supplementary video (Online Resource 2). Section 0 illustrates the capabilities of the robot by showing data of a possible device test as well as an analysis of the accuracy of the simulation robot.

### Pre-Processing

Our pre-processing algorithm proved able to generate the required motor setpoints from recorded heart motion trajectories. The numerical computation is fast, reliable and accurate. According to the Simulink model, the Stewart platform moves for all tested heart motions within the knee joint limits between 63 and 140 degrees for bending and stretching the legs, respectively (cf. Table 1). The displacements required to simulate these motions are well within the physical limits of the Stewart platform’s workspace radius of 29 mm and the limit of 19 degrees of maximal deflection angle.

**Table 1:**
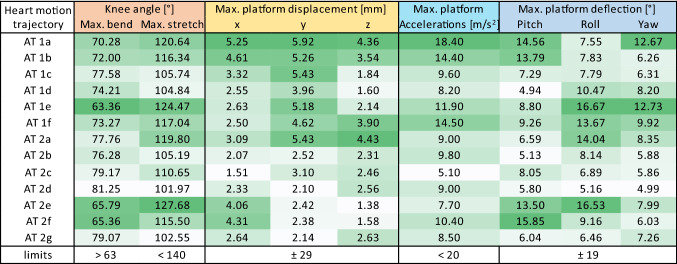
Shows the maximum values of 13 simulated heart motions using the Simulink model.

### Post-Processing

**Figure 5:**
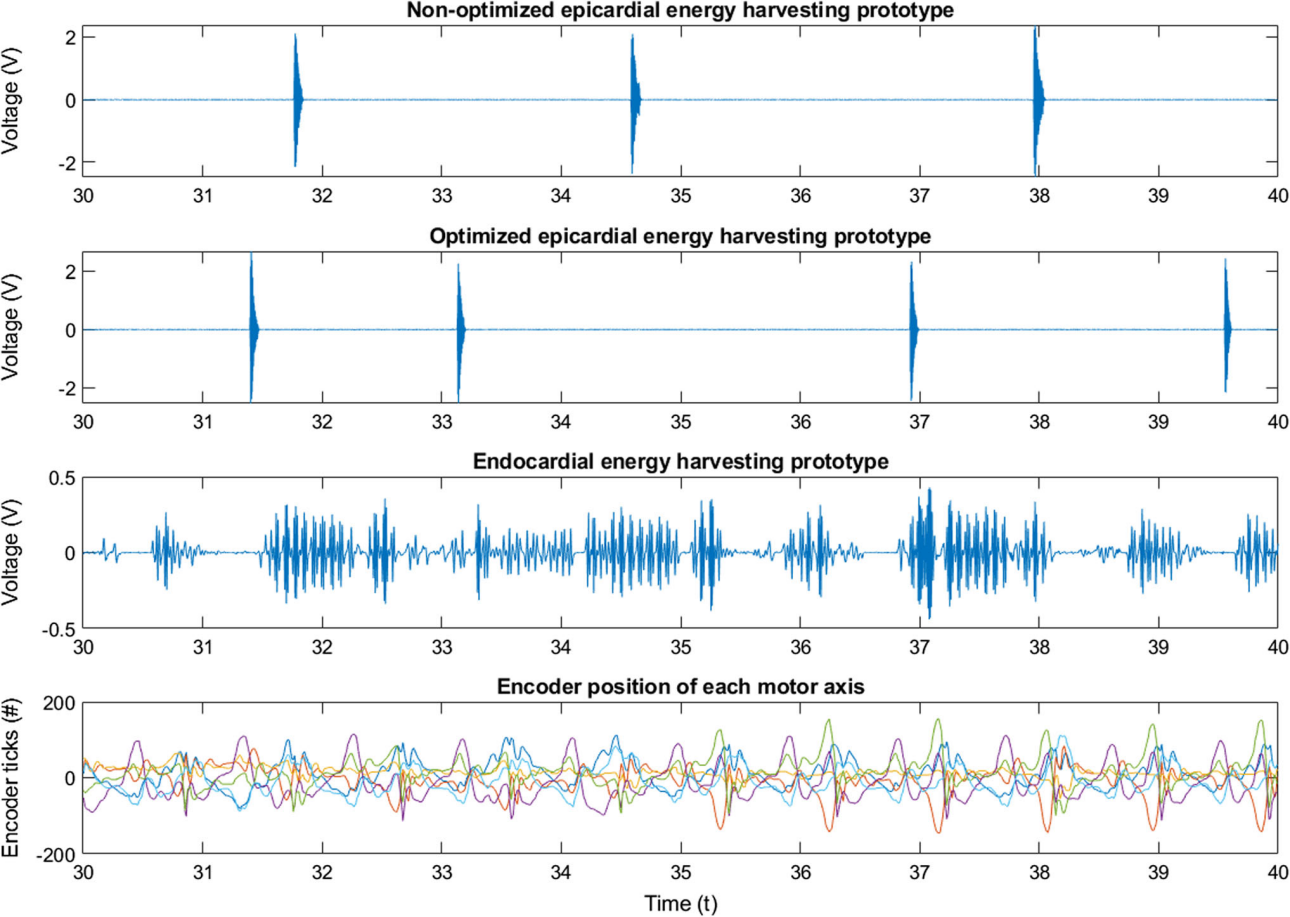
Illustrates 10 s segments of the output signals acquired from the three different energy harvesting devices. Furthermore, the motors’ angular positions $enc\left( t \right)$ are indicated in six different colors in the bottom panel.

The electrical voltage signals from all three devices were recorded along with the encoder positions $enc\left( t \right)$ over a period of 60 s. The 10 s-long segments in Fig. 5 depict the different voltage outputs of different devices excited by the same heart motion.

**Figure 6:**
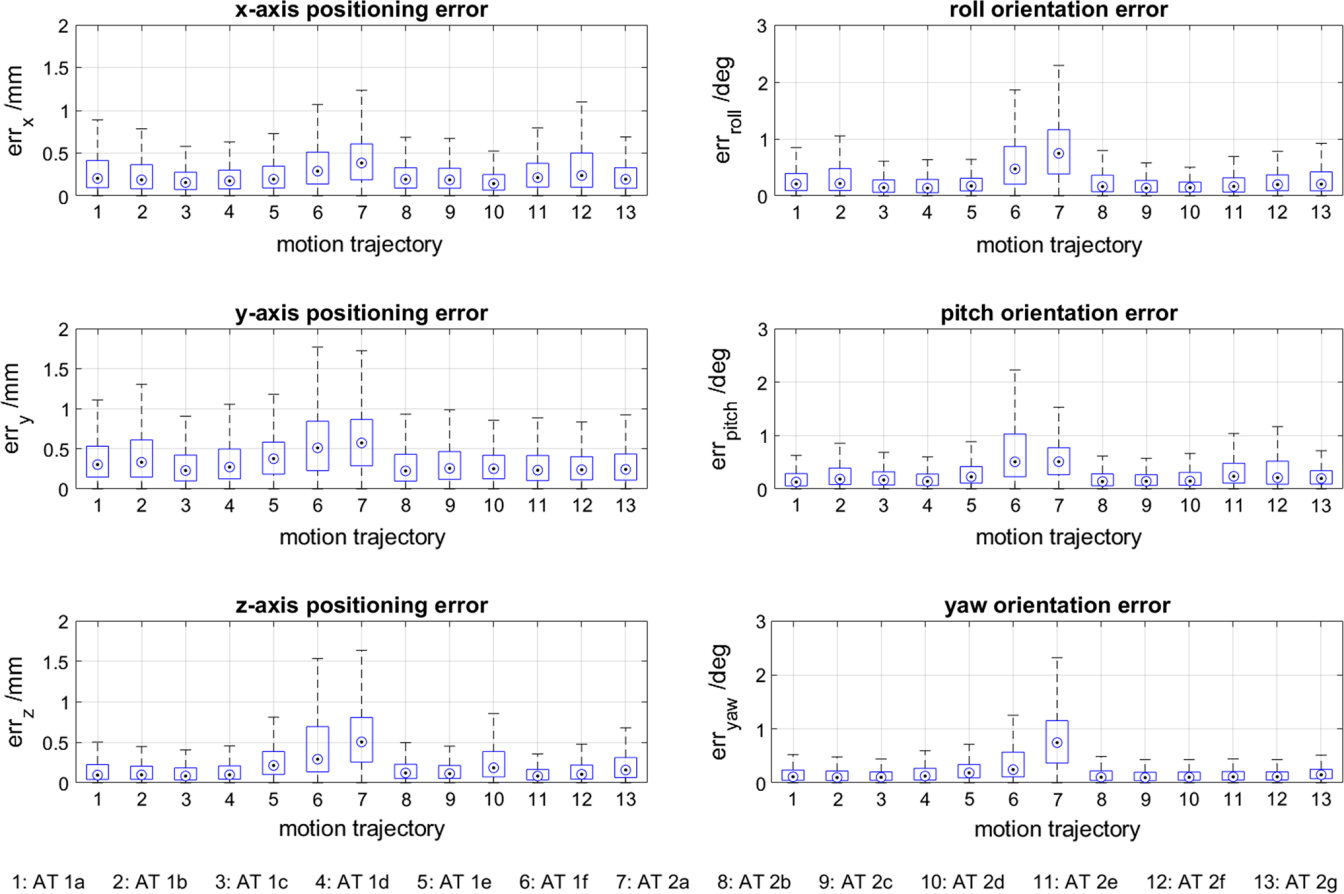
Illustrates the positioning errors $err_{o} \left( t \right)$ in x-, y- and z-direction and orientation errors $err_{\phi } \left( t \right)$ about x-, y- and z-axis (roll, pitch and yaw) for 13 different heart motion trajectories.

The Stewart platform’s encoder values, generated by reproducing 13 different heart motions, were compared to the set point trajectories $o\left( t \right)$ and attitudes $\phi \left( t \right)$ of each individual heart motion. As illustrated in Fig. 6, the Stewart platform’s positioning and orientation accuracy (relative to the desired position) depends on the heart motion trajectory. The absolute mean positioning error (± standard deviation) in x-, y- and z-direction is 0.21 ± 0.06, 0.31 ± 0.11 and 0.17 ± 0.12 mm, respectively. The absolute mean orientation error around the x-, y- and z-axis (roll, pitch and yaw) is 0.24 ± 0.18°, 0.23 ± 0.13° and 0.18 ± 0.18°, respectively.

## Discussion

We introduce a robot that mimics physiological heart motion trajectories and attitudes in an accurate and repetitive manner. This allows reproducing the trajectory of any point of the heart that was previously obtained by imaging techniques (e.g. magnetic resonance tomography, computer tomography or echo-cardio-graphy) or that was artificially generated. Furthermore, implants (e.g. the leadless pacemaker Micra™ AV, Medtronic, USA) or custom-built inertial measurement units may also provide useful input data.

### Technical Considerations

The Stewart platform considers each input as a fixed target heart motion trajectory. Therefore, the robot’s PID controller tries to match the targeted values as good as possible. However, we are aware that an additional load attached to a certain point on the heart *in-vivo* may affect the heart’s motion trajectory. The implementation of an appropriate compensation mechanism to generate a loaded trajectory for *ex-vivo* purposes from a recorded unloaded *in-vivo* trajectory is beyond the scope of this study. Indeed, it would require a complex mechanical model of the heart wall, which would have to be solved numerically e.g. via the finite element method. This heart wall model would further have to be fed with material parameters which may be strongly dependent on a patient’s health. Finally, it would appear that the recorded trajectory and the 3D geometry of the model would have to originate from the same heart for the compensation to make any sense. For sufficiently small loads however, it seems reasonable to assume that the impact on the trajectory of the attachment point will be negligible. A recorded unloaded trajectory will therefore still be a useful input to conduct *ex-vivo* experiments with the Stewart platform. For example, a pacemaker lead or a tiny energy harvesting device are unlikely to noticeably affect the motion of the heart. On the contrary, if the load becomes more important to the point of perturbing the natural motion of the heart, the recording of a loaded *in-vivo* trajectory would be required to feed the robot with meaningful data. However, attaching an object to the myocardium that would significantly change heart dynamics is questionable. Experiments within a physiological fluid represent another scenario which would require the robot to perform more work. To this end, the robot would have to be mounted upside down, such that the end effector can be immersed in the fluid. While this has not been demonstrated in the present study, there is nothing that prevents the robot from being operated upside down. To which extent the robot will be able to reproduce the recorded trajectory when the end effector is moving in a fluidic environment will depend on the viscosity. Eventually, PID control parameters need to be adjusted to overcome additional forces created by the submerged end effector. If this measure does not provide the desired effect, structural changes would become necessary, e.g. enhanced legs, joints and/or motors.

As mentioned above, there are many external influencing factors that can affect the trajectory and attitude during *ex-vivo* experiments. For our specific experiments, the PID controller values were adjusted to account for the weight of the end effector and the additional load of a device. This is a crucial measure to reduce positioning errors to a minimum. Considering the high accelerations that need to be reproduced for mimicking heart motions, the absolute mean errors reported in section 0 are very small. Due to these small errors, their standard deviations appear comparatively big, when in fact the deviations are similarly small. In addition to that, Fig. 6 shows that motion trajectories 6 and 7 have noticeably higher error values than the rest of the trajectories. This is due to the fact that these two trajectories comprise high acceleration spikes, which were harder to reproduce with the given set of PID controller values. To make the results comparable for this study, it was decided not to adjust PID values for each individual trajectory, which in practice should be done to get the most accurate results. Since the heart motion is a repeating motion trajectory, one could envision to improve accuracy further by implementing a machine learning-based controller. This would also facilitate the process of finding new best controller parameters for experiments with different load scenarios.

The end effector of the Stewart platform has a workspace with the shape of a sphere with a radius of 29 mm. Furthermore, it can tilt by ± 19 degrees around all axes. However, within said workspace, it is not possible to achieve all combinations of displacement and tilting of the platform. For example, when its center is positioned on the outer shell of the sphere, the platform cannot be tilted by the full 19 degrees anymore. Consequently, heart motions consisting of trajectories $o\left( t \right)$ and attitudes $\phi \left( t \right)$ typically cannot at the same time use the full translational and the full rotational freedom. Therefore, before a heart motion trajectory is used on the test bench, it will be simulated in-silico to check whether the target trajectory fulfills the workspace constraints. To use the given workspace most efficiently, every heart motion will be centered with respect to the origin of the robot’s coordinates. Furthermore, the initial orientation of the device is set by the mounting frame on top of the end effector and allows the platform to start at a neutral attitude.

The knee joints provide a large range of motion, which is sufficient to reproduce physiological heart motions including all heart motions from our current data collection. Currently, the maximal knee bending angle is given by design at 63 degrees due to the physical restriction of the joint. Also, we decided to limit the knee stretching angle to a maximum of 140 degrees to avoid the risk of overstretching the leg structure. Heart motions that exceed the current joint limits, even after centering the motion to the workspace programmatically, will necessitate a redesign of the joints or leg lengths.

The exemplary motion experiment was conducted with three different energy harvesting devices, which were exposed to the same heart motion. This illustrates the very different nature of the prototype iterations. The purpose of this experiment was to demonstrate the use of our robot and not to judge on design performance of the harvesting devices. This has been reported in more detailed studies,^21^,^5^ where the energy harvesting prototypes were tested with the Stewart platform.

### Implications for Biomedical Device Testing, Training and Animal Welfare

Medical devices that need to be developed and tested can repetitively be investigated on the bench using our robot. This allows exposing different device iterations during a development cycle always to the same motion pattern. Thus, it is easier to understand and quantify the effect of a design change on the device’s behavior.

A robotic platform that can mimic heart motions can be used for various purposes, but its use depends on the device intended to be tested. For instance, the development of batteryless pacemakers harvesting energy from the cardiac wall motion will benefit substantially from such platforms. Batteryless devices are expected to enter the market in just a few years (e.g. the ALPS developed by Cairdac SA (France)). Their functionality will heavily depend on the local movement of the endocardial attachment site. Moreover, a robotic platform may help investigating the anchoring stability of contemporary leadless pacemakers. For instance, atrial leadless pacemakers are under development.^15^ A stable atrial anchoring of such a device is crucial but difficult to develop given the fragile nature of the right atrial wall. Thus, *ex-vivo* studies on a robot may allow minimizing traumatic effects induced by the sharp and flexible anchors. Finally, there is increasing awareness that certain pacemaker leads undergo midterm failure, that may be related to a complex interaction of their stiffness and the local forces onto the myocardium.^7^ Such complex effects may be studied in a controlled setting using a complex robotic platform as presented by the authors. The complex interaction of a lead and the endocardial wall may be studied in much more detail compared e.g. to simple repetitive bending tests of a lead.

In contrast, a hexapod robot may offer little value when it comes to more hemodynamically oriented testing of artificial heart valves. For this purpose, mock-circulation setups are more appropriate. While standardized flow-loops and fatigue testers have traditionally been used for many years, there is—similar as we intend with our development—an increasing trend towards more patient-specific testing.^9^

Besides device testing, the platform may be used for training purposes of cardiac surgeons. Beating heart surgery requires significant procedural expertise and training simulators for off-pump surgery have been proposed.^12^ Our robot is able to simulate highly complex 3D motions of the epicardial surface. This could be used for the training of coronary anastomoses during coronary artery bypass grafting—the procedure could just be performed on a small piece of tissue directly on the simulator.

Finally, such robotic platforms may offer great potential to reduce the number of animal trials and early clinical in-human trials and thereby serves as a valuable adjunct with respect to 3R and animal welfare efforts. For example, the 13 heart motions that have been utilized in this study were obtained in a previously conducted study.^21^ After that, these datasets have repeatedly been used to investigate other energy harvesting concepts for many years^5^,^22^,^23^ without demanding additional *in-vivo* trials to prove a concept.

## Conclusion

This study presents a novel robotic approach to reproduce physiological heart motions with high accuracy and repeatability to study their effect on implantable cardiac devices. This may benefit the device development process and offer the potential to increase safety and quality of next-generation implantable cardiac devices. Most importantly, our approach allows re-using heart motion data repeatedly without sacrificing the life of animals, thereby promoting the 3R principles.

## Supplementary Information

Below is the link to the electronic supplementary material.

Supplementary file1 (MP4 367 kb)

Supplementary file2 (MP4 20727 kb)

## Data Availability

Heart motion experiment were performed based on heart motion trajectory datasets that were previously acquired by Refs. 20, 21.

## Code availability

Pseudocode of the inverse kinematic algorithm is provided in “Pre-Processing” section. The source code with the implementation of this algorithm is custom-tailored to the controller hardware used in this work. Thus, it is not provided in this publication but will be available upon request.

## Research Involving Animal Studies

No animal studies were carried out by the authors for this article.
